# Nanoscaffold-based 3D human liver spheroids for predictive hepatotoxicity screening of antimalarial compounds from the global health priority box

**DOI:** 10.1186/s13071-026-07324-1

**Published:** 2026-03-23

**Authors:** Lina Wu, Driton Vllasaliu, Diana Ayoola Mabayoje, Adam Aspinall, Bahijja Tolulope Raimi-Abraham

**Affiliations:** 1 School of Cancer and Pharmaceutical Sciences, Faculty of Life Sciences and Medicine, Institute of Pharmaceutical Science, King’s College London, Franklin-Wilkins Building, 150 Stamford Street, London, SE1 9NH UK; 2 Bart’s Health NHS Trust, London, UK; 3 Medicines for Malaria Venture (MMV), Rte de Pré-Bois 20, 1215 Geneva, Switzerland

**Keywords:** 3D liver spheroids, Hepatotoxicity screening, Antimalarial drug development

## Abstract

**Background:**

Drug-induced liver injury (DILI) remains a significant barrier to the safe and efficient use of antimalarial medicines. Many promising compounds fail in late-stage development or post-marketing owing to unforeseen toxicity, particularly DILI. Incorporating a predictive hepatotoxicity assessment is therefore critical to reduce clinical risk and development costs. The Medicines for Malaria Venture (MMV) Global Health Priority Box (GHPB) provides a library of compounds with demonstrated or potential antimalarial activity, yet their hepatotoxicity risk remains poorly defined. Our early work developed and validated a nanoscaffold-based three-dimensional (3D) liver spheroid platform. Here, we apply this validated model for the first time to assess the hepatotoxicity of clinically used and candidate antimalarial GHPB compounds.

**Methods:**

Our validated nanoscaffold-based 3D liver spheroid platform was used to evaluate the hepatotoxicity of approved antimalarial drugs of known liver toxicity (quinine, primaquine, amodiaquine, sulfadoxine/pyrimethamine, and artemisinin) and six GHPB candidate compounds. Half maximal inhibitory concentration (IC_50_)-derived data from the approved antimalarial drugs were used to generate a reference framework on the basis of established DILI classifications, which was then applied to categorize the GHPB candidate compounds relative to hepatotoxic risk.

**Results:**

Our nanoscaffold-based 3D liver spheroid platform accurately reproduced the known DILI rankings of the approved antimalarials, confirming its predictive validity. Using these referenced IC_50_-derived profiles, candidate compounds from the GHPB were classified into distinct hepatotoxicity categories, ranging from low: MMV1167451 (compound 01) and MMV020192 (compound 02), moderate: MMV1797658 (compound 03) and MMV1435700 (compound 04), to high: MMV006344 (compound 05) and MMV006931 (compound 06) risk, demonstrating the model’s capacity to support early-stage animal-free antimalarial hepatotoxicity screening.

**Conclusions:**

This study demonstrates the translational application of a validated nanoscaffold-based 3D human liver spheroid model for antimalarial drug in vitro hepatotoxicity assessment. By establishing a reference framework from clinically approved antimalarials and applying it to candidate compounds from the MMV GHPB, our platform enabled early classification of hepatotoxicity risk using a human-relevant, non-animal method. The findings support the integration of advanced 3D in vitro systems into antimalarial drug discovery pipelines to improve safety prediction, reduce reliance on animal testing, and accelerate the development of safer, more effective antimalarial therapies.

**Graphical Abstract:**

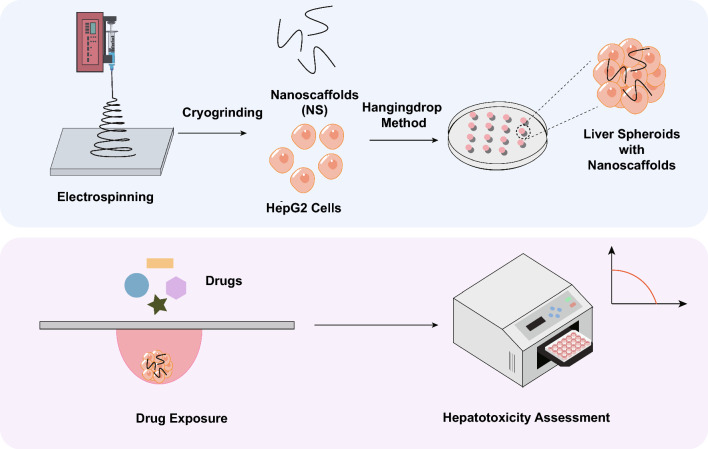

**Supplementary Information:**

The online version contains supplementary material available at 10.1186/s13071-026-07324-1.

## Background

Malaria is one of the oldest infectious diseases that still has significant global impact to date. According to the World Health Organization (WHO) Malaria report 2024, 263 million new cases occurred in 2023, causing 597,000 deaths, with most of the deaths being among children aged under 5 years [1]. Malaria is caused by several species of malaria parasites, including *Plasmodium falciparum*, *Plasmodium vivax*, *Plasmodium ovale*, and *Plasmodium malariae*, with *P. vivax* being the most widespread malaria parasite and *P. falciparum* causing the most morbidity and mortality [2]. Effective prevention, early diagnosis, and prompt treatment of malaria can prevent severe complications and transmission [3]. The WHO recommends antimalarial medications as the primary treatment and prevention for malaria infections [1].

While antimalarial drugs are crucial for the treatment and prevention of malaria, liver injuries caused by hepatotoxic drugs, referred to as drug-induced liver injury (DILI), remain a significant challenge in both the development of new drugs and the clinical use of antimalarial medications. Clinical reports have documented cases of life-threatening liver failure and fatalities associated with certain antimalarial medications [4–6]. For example, amodiaquine (AQ) is a potent antimalarial drug and is widely prescribed in endemic areas. However, owing to its side effects, such as severe hepatotoxicity and agranulocytosis, AQ was withdrawn from general use for malaria prophylaxis in 1986 [7]. In addition, sulfadoxine/pyrimethamine is known to cause liver toxicity, including hepatic granulomas, mixed cholestatic-hepatocellular hepatitis, acute hepatic necrosis, and chronic hepatitis [4, 5]. DILI not only has profound negative impacts on public health as DILI sufferers often experience poor physical and psychological health, resulting in a low quality of life [8], but also imposes significant socioeconomic burdens. The UK Government has committed £7.4 million to improve access to antimalarial drugs and diagnostic tests, aiming to reach over 50 million people by 2027 [9], and reducing the DILI risks could optimize the return on antimalarial research funding.

The ‘Global Health Priority Box’ (GHPB) assembled by the Medicines for Malaria Venture (MMV) represents a particularly valuable resource for antimalarial drug discovery [10]. MMV collaborates with academic institutions, pharmaceutical companies, and regulatory agencies to establish standardized compound libraries and screening guidelines [11]. The GHPB includes a curated collection of promising compounds with known antimalarial activity as well as reference compounds with established profiles [12]. While the antimalarial efficacy of these compounds has been investigated, their pharmacological and toxicological properties, particularly hepatotoxicity, have not been fully characterized. Incorporating advanced liver models into the screening of GHPB compounds may offer a valuable approach for the early identification of potential hepatotoxic liabilities, thereby supporting the development of safer antimalarial therapies.

Establishing a bio-predictive model to screen drug candidates for liver toxicity is key to developing safer antimalarial (and other) therapies. Currently, the preclinical testing of new drugs primarily relies on monolayer two-dimensional (2D) cell cultures and animal models as required by the US Food and Drug Administration (FDA), which remain key experimental methods for investigating DILI [12]. However, owing to the biological differences between animals and humans, drugs that cause severe DILI in humans often do not show obvious hepatotoxicity in animal models [13]. A study comparing the toxicity of 150 compounds between human and animal models found that nonrodent and rodent animal models could predict only about 63% and 43% of DILI cases in humans, respectively [14]. Furthermore, ethical concerns have increasingly restricted the use of animal models. In this regard, the FDA took a major step in 2022 by approving the ACT 2.0 Act, which eliminated the requirement for animal testing before new drug approvals [15], and introduced ACT 3.0 in 2024, which mandates the development of processes to support nonclinical testing methods for drug development that do not involve the use of animals [16]. Therefore, there is a growing need for substitutes for animal models.

For over a century, 2D cell models have been widely used as in vitro models in drug discovery research [17]. While 2D cell cultures have contributed significantly to the understanding of various biological and disease processes, they often fail to replicate the complex in vivo microenvironment [18, 19]. For example, 2D cultured cells will rapidly dedifferentiate and lose liver-specific functions, such as albumin secretion and drug-metabolizing enzyme activity within hours in vitro [20].

In addition, the expression of drug metabolizing enzymes, mainly the cytochrome P450 (CYP) enzyme family, shows significant variability among individuals [21]. Further, 2D cell models always fail to detect patient-specific toxicities resulting from gene polymorphisms owing to the lack of genetic variation, making them unsuitable for personalized toxicity prediction [22].

This limitation has led to a growing interest in developing and employing three-dimensional (3D) cell culture models in drug development. Unlike 2D cultures, the 3D culture method allows cells to grow in all dimensions, enabling them to interact with the surrounding extracellular environment and better mimic human tissue [23]. Moreover, the activity of drug-metabolizing enzymes in 3D cell culture models could be tuned, which would enable genotype-specific customization and personalized hepatotoxicity prediction [24, 25].

Spheroids are self-assembled 3D cell aggregates, which are increasingly being used in drug development [26–28]. Conventional spheroids are typically scaffold-free. However, in the human body, cells grow within the extracellular matrix (ECM), which not only provides support for cellular structure but also enables the transport of essential nutrients, functional activity, and metabolic transport [29, 30]. Therefore, incorporating scaffolds which could mimic the natural ECM in spheroids may generate a more physically relevant model for in vitro studies. In our previous work, we established an in situ self-assembled liver spheroid platform using synthetic electrospun polycaprolactone (PCL) nanoscaffolds [24]. We have successfully demonstrated that the presence of nanoscaffolds significantly enhances spheroid performance, including improved albumin and urea secretion, and enhanced drug-metabolizing enzyme activity, and validated its potential for drug-induced liver toxicity screening [24]. However, this initial model primarily focused on foundational hepatic functionalities, with limited exploration of in-depth drug metabolism dynamics and no experimental validation of its translational applicability.

In the work presented here, we address these gaps by further refining the metabolic properties of the spheroids, including the expression and inducibility of major drug-metabolizing enzymes (i.e., CYP3A4, CYP1A2, and CYP2B6) and employing the model for both commercially available antimalarials and candidate compound hepatotoxicity screening. Clinically approved antimalarial drugs with known DILI profiles were first assessed to generate IC_50_-derived benchmarks for toxicity classification, which were then applied to six candidate compounds from the GHPB. This work addresses a critical gap in predictive toxicology and provides a translational tool to enhance the safety and efficiency of global health drug discovery pipelines.

## Methods

### Materials

Polycaprolactone (PCL), fetal bovine serum (FBS), trypsin–EDTA solution, Minimum Essential Medium Eagle (MEME), nonessential amino acid solution (NEAA), L-glutamine solution, doxorubicin, and antimalarials (quinine, primaquine, artemisinin, amodiaquine, sulfadoxine, and pyrimethamine) were purchased from Sigma-Aldrich (UK). Oxoid phosphate-buffered saline (PBS) tablets (Dulbecco A), carbamazepine, LIVE/DEAD cell imaging kit (488/570), fixative solution, AlamarBlue cell viability reagent, albumin human ELISA kit, FAK [pY397] ELISA kit, Superscript IV Vilo mix, and SYBR Green were purchased from ThermoFisher (UK). CellTiter-Glo 3D Cell Viability Assay reagent and the P450-Glo CYP1A2, 2B6, and 3A4 Assays were purchased from Promega (UK). Anti-integrin beta 1 antibody [P5D2] (Alexa Fluor^®^ 488) was purchased from Abcam (UK). The GHPB antimalarial compounds (MMV 01–06) were kindly provided by MMV. The chemical structural information is detailed in Table 1.

**Table 1:** Medicines for Malaria Venture (MMV) compounds used in this study

ID	MMV ID	Formula	Area of research
01	MMV1167451	C_28_H_28_N_6_OS	Drug-resistant malaria
02	MMV020192	C_27_H_24_FN_3_O_3_	
03	MMV1797658	C_21_H_26_N_4_	
04	MMV1435700	C_18_H_17_N_3_O_4_S_2_	
05	MMV006344	C_21_H_25_ClN_4_OS	
06	MMV006931	C_30_H_28_N_4_O_3_S	

*MMV*, Medicines for Malaria Venture

### Preparation and characterization of polycaprolactone (PCL) nanoscaffolds

PCL nanoscaffolds were generated as described in previous work [24]. Briefly, PCL (Sigma-Aldrich, UK) was dissolved in acetone at a final concentration of 10% (w/v). Electrospinning was performed at 19 kV/15 cm with a 19-gauge needle (inner diameter = 0.686 mm, outer diameter = 1.607 mm). The electrospinning experiments were performed at an ambient temperature of 20–22 °C and humidity of 40–50%. The resultant nanofibers were air-dried overnight to remove the residual solvents. Nanoscaffolds were generated by mechanical grinding after freezing by liquid nitrogen [24].

The surface characteristics of the electrospun PCL nanofibers were characterized using a scanning electron microscope (SEM; Phenom Pro, UK). The nanofibrous membrane/nanoscaffolds were fixed onto a specimen stub and sputter-coated with a 10-nm gold layer to enhance conductivity. Quantitative analysis was performed using ImageJ software. A representative dataset of 100 randomly selected fibers was measured to calculate the mean diameter with the associated standard deviation. The diameter distribution profile was statistically analyzed and visualized through a frequency distribution histogram generated using GraphPad Prism 10 (San Diego, CA, USA).

### Cell culture and spheroid generation

The human hepatocellular carcinoma cell line (HepG2) was purchased from Abcam (UK). The HepG2 cells were cultured in a complete MEME medium (10% FBS, 1% NEAA, 1% L-glutamine, and 1% penicillin–streptomycin). HepG2 spheroids with and without nanoscaffolds were generated by the hanging drop method as previously described [24]. Briefly, trypsinized cells were pelleted at 1000 rpm for 3 min, then resuspended in a complete tissue culture medium. Cells were counted using a hemacytometer, and the concentration was adjusted to 2.5 × 10^4^ cells/ml. Then, 20-μl drops containing 500 cells were deposited onto the bottom of the petri dish lid. The lid was inverted onto the PBS-filled bottom chamber and incubated at 37 °C/5% CO_2_/95% humidity. The cell culture medium was changed every 2 days.

For the generation of spheroids with nanoscaffolds, the nanoscaffolds were sterilized with an ultraviolet (UV) lamp for 12 h and then mixed with HepG2 cells. Spheroids with nanofibers were generated and cultured with the same method as the ones without nanofibers. For ease, spheroids without nanoscaffolds and spheroids with 0.005% w/v nanoscaffolds will be referred to as S0 and S-NS, respectively.

The generation process of HepG2 spheroids was observed with a phase contrast microscope on different days. Spheroids were stained with a LIVE/DEAD Cell Imaging kit according to the manufacturer’s instructions. First, the spheroids were collected from the drops and transferred into a 96-well plate. Subsequently, equal volumes of imaging reagents were added to the spheroids and incubated for 30 min at 37 °C in the dark. After incubation, the stained spheroid samples were analyzed by fluorescence microscopy under the excitation/emission wavelengths of 488/515 nm for live cells and 570/602 nm for dead cells.

### Drug-metabolizing enzyme activity

Rifampicin, quinine, and carbamazepine were used as inducers of CYP3A4, CYP1A2, and CYP2B6, respectively. To start, 2D cells, S0 and S-NS (day 5) were treated with rifampicin (50 μM), quinine (30 μM), and carbamazepine (50 μM) for 72 h, respectively. The drug-metabolism enzyme activity was evaluated using P450-Glo™ CYP1A2, CYP2B6 2B6 and CYP3A4 Assay kits according to the manufacturer’s protocol. Briefly, HepG2 spheroids were collected and washed with PBS twice and incubated with 200 μl enzyme substrate (3 μM luciferin-IPA, 6 μM Luciferin-1A2, and 3 μM Luciferin-2B6) for 1 h at 37 °C. After incubation, 25 μl of supernatant was transferred to a white opaque 96-well plate, and 25 μl detection reagent was added. The mixture was incubated for 20 min at room temperature, and luminescence was measured using a plate reader. Data were normalized to cell number as measured by the CellTiter-Glo^®^ assay.

### Quantitative reverse transcription PCR (RT-qPCR)

Total mRNA was isolated from a pool of 300 spheroids for each studied compound and control using TRIzol. The concentration of isolated mRNA was determined using a NanoDrop 1000 Spectrophotometer. The cDNA High-Capacity Archive Kit was used for the reverse transcription of 1 μg of total mRNA per sample. qPCR was performed according to the manufacturer’s instructions (Applied Biosystems, Studio 7) with SYBR green master mix. Target mRNA levels were presented relative to the amount of the housekeeping gene (*GAPDH*). Primer sequences are listed in Additional File 1 (Table S1).

### Albumin and urea secretion detection

Albumin and urea secretion levels in HepG2 spheroids, both with and without nanoscaffolds, were measured using an albumin human ELISA kit (Invitrogen) and a urea assay kit (Abcam), following the manufacturer’s instructions. Absorbance readings were taken at a wavelength of 450 nm.

### Immunofluorescence

The spheroids were collected and washed with cold PBS three times and subsequently fixed with 4% formaldehyde for 30 min at room temperature, followed by permeabilization with 0.2% Triton-X 100 for 15 min. The spheroids were then blocked with 5% BSA in PBS for 60 min at room temperature. Conjugated antibodies were diluted in 5% BSA according to the manufacturer’s instructions and incubated with the spheroids overnight at 4 °C. Spheroids were finally mounted with ProLong™ Gold Antifade Mountant with DNA stain DAPI onto a glass microscope slide. After incubation, the fluorescence images were obtained by fluorescence microscopy under the excitation/emission wavelengths of 488 nm and the mean fluorescence intensity was analyzed by ImageJ.

### pFAK level

In total, 20 spheroids, both with and without nanoscaffolds, were collected on days 3, 5, 7, and 9. The pFAK levels were quantified using a Human FAK [pY397] ELISA kit, following the manufacturer’s protocol. Absorbance was measured at a wavelength of 450 nm using a plate reader.

### Drug penetration

The spheroids with and without nanoscaffolds were cultured for 5 days and incubated with 2 μM doxorubicin at 37 °C in the dark for 24 h. After incubation, the culture medium was discarded, and the spheroids were washed three times using PBS. Images were taken by fluorescence microscopy under the excitation wavelength of 488 nm.

### Drug screening

The in vitro hepatotoxicity of selected antimalarial drugs and MMV compounds was determined on HepG2 spheroids after 5 days of culture, with 2D cultured HepG2 and spheroids without nanoscaffolds used as control. Cell viability of 2D cells and spheroids was determined by Alamar blue and Celltiter Glo assays, respectively. The details of the compounds used are listed in Additional File 1 (Table S2). The IC_50_ values were calculated by Prism 10 software.

### Statistical analysis

Data expressed are representative of at least three independent experiments (*n* = 3) in triplicate and represented as mean ± standard error. A Student’s *t*-test was used to discern the statistical difference between the two groups (S0 and S-NS) and a one-way analysis of variance (ANOVA) test was used to discern the statistical difference between three or more groups (2D, S0, and S-NS). A probability value (*P*) of less than 0.05 was considered statistically significant. Graphs and statistical analysis were performed using GraphPad Prism 10 (GraphPad Software, San Diego, CA, USA).

## Results

### The generation of nanoscaffolds and spheroids with and without nanoscaffolds

**Fig. 1:**
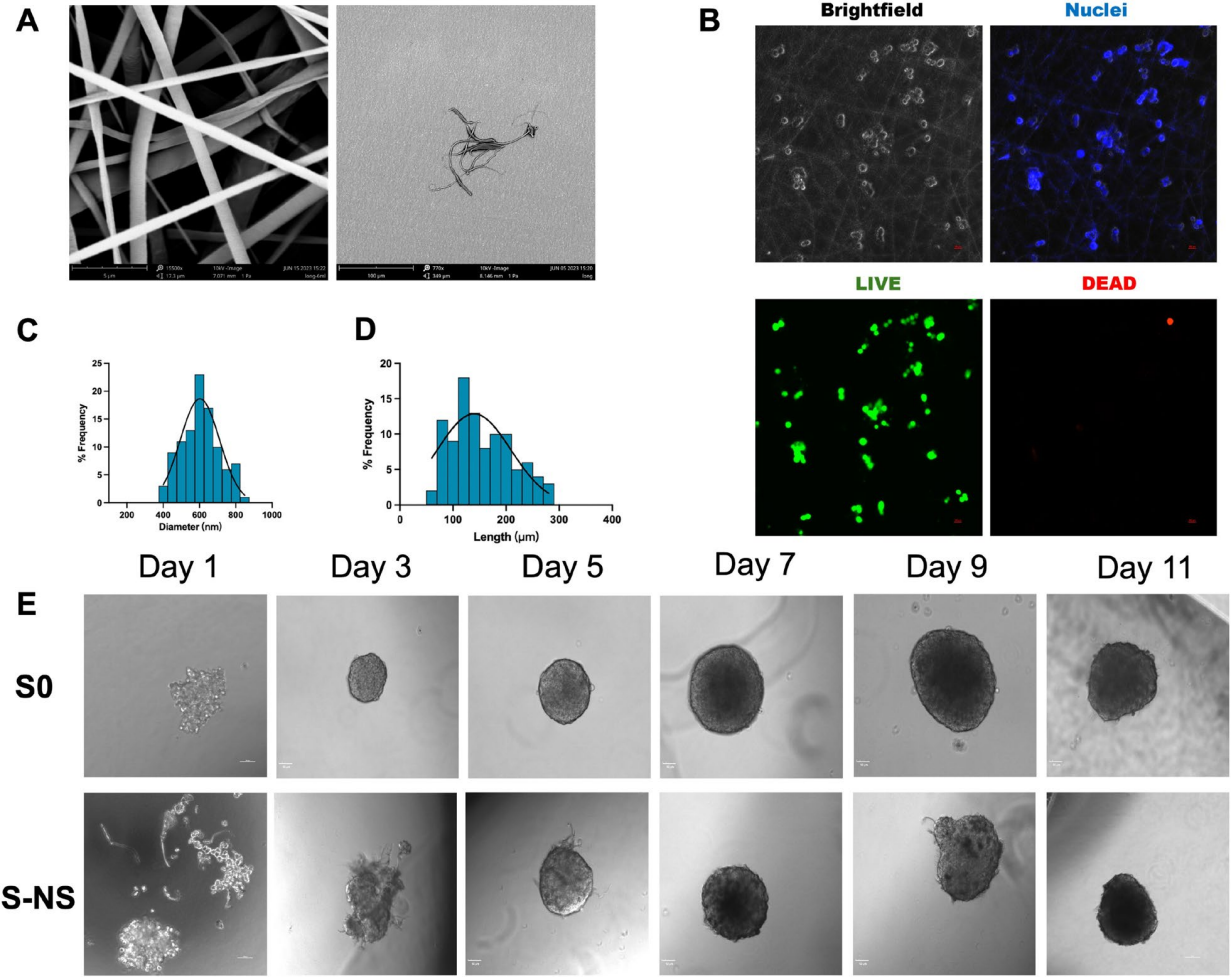
**A** SEM of nanofiber mesh (left) and nanoscaffolds (right). Scale bars: 5 μm (left) and 100 μm (right). **B** Attachment of 2D HepG2 cells on PCL nanofiber. Scale bar: 100 μm. **C** Diameter distribution of nanoscaffolds (*n* = 100). **D** Length distribution of nanoscaffolds (*n* = 100). **E** Generation of spheroids with and without nanoscaffolds. Objective: 20×, Scale bar: 50 μm. SEM, scanning electron microscopy; PCL, polycaprolactone; S0, spheroids without nanoscaffolds; S-NS, spheroids with nanoscaffolds

Electrospun PCL nanofibers were successfully generated, and nanoscaffolds were then obtained by cryogrinding. SEM was employed to observe the morphology of the nanoscaffolds. Figure 1A shows that PCL nanofibers showed a nanoscale fibrous structure. To assess the biocompatibility of the PCL nanofibers, we observed cell attachment on the PCL nanofibers. Single layer electrospun PCL nanofibers were directly deposited onto the glass slides for cell culture to avoid obstructing the field of view. As shown in Fig. 1B, after 4 days of seeding, HepG2 cells were randomly distributed on the PCL nanofibers and maintained a normal cell morphology, indicating that the cells could adhere to the nanofiber effectively. In addition, live/dead fluorescence staining indicated good cell viability (green fluorescence) with few dead cells observed in the view field (red fluorescence), further confirming the low cytotoxicity of the PCL nanofibers. These results indicated that the PCL nanofibers provided a favorable environment for cell growth and exhibited good biocompatibility, which highlights their potential for their applications in tissue engineering scaffolds.

Single nanofibers were obtained after cryogrinding and used as nanoscaffolds for the generation of scaffold-based nanoscaffolds. The nanofibrous structure remained in the nanoscaffolds, indicating that the grinding process did not affect the morphology of the nanofiber as previously observed [24]. The diameter distribution of nanoscaffolds ranged from 400 to 900 nm, with an average diameter of 605.645 ± 103.97 nm (Fig. 1C). The length distribution of nanoscaffolds was relatively broad (between 50 and 300 µm), with the average length of nanoscaffolds approximately 154.81 ± 56.17 µm (Fig. 1D).

As shown in Fig. 1D, spheroids without nanoscaffolds (S0) and with nanoscaffolds (S-NS) with well-defined perimeters were formed within 5 days after seeding, with a spherical and intact morphology remaining stable for 11 days. Compared with S0, which could form into a spheroid by day 3 (Fig. 1E), cells in S-NS conditions initially attached to the nanoscaffolds (day 1 to day 3), followed by the establishment of cell–cell interactions and eventual spheroid formation. The spheroid sizes remained below 500 µm throughout the 9-day period, with a maximum diameter of 397 µm. The ability of the spheroid diameter to be maintained at < 500um ensures the adequate diffusion of oxygen and other nutrients to the core of the spheroids in order to avoid necrosis.

### The influence of nanoscaffolds on cell viability

**Fig. 2:**
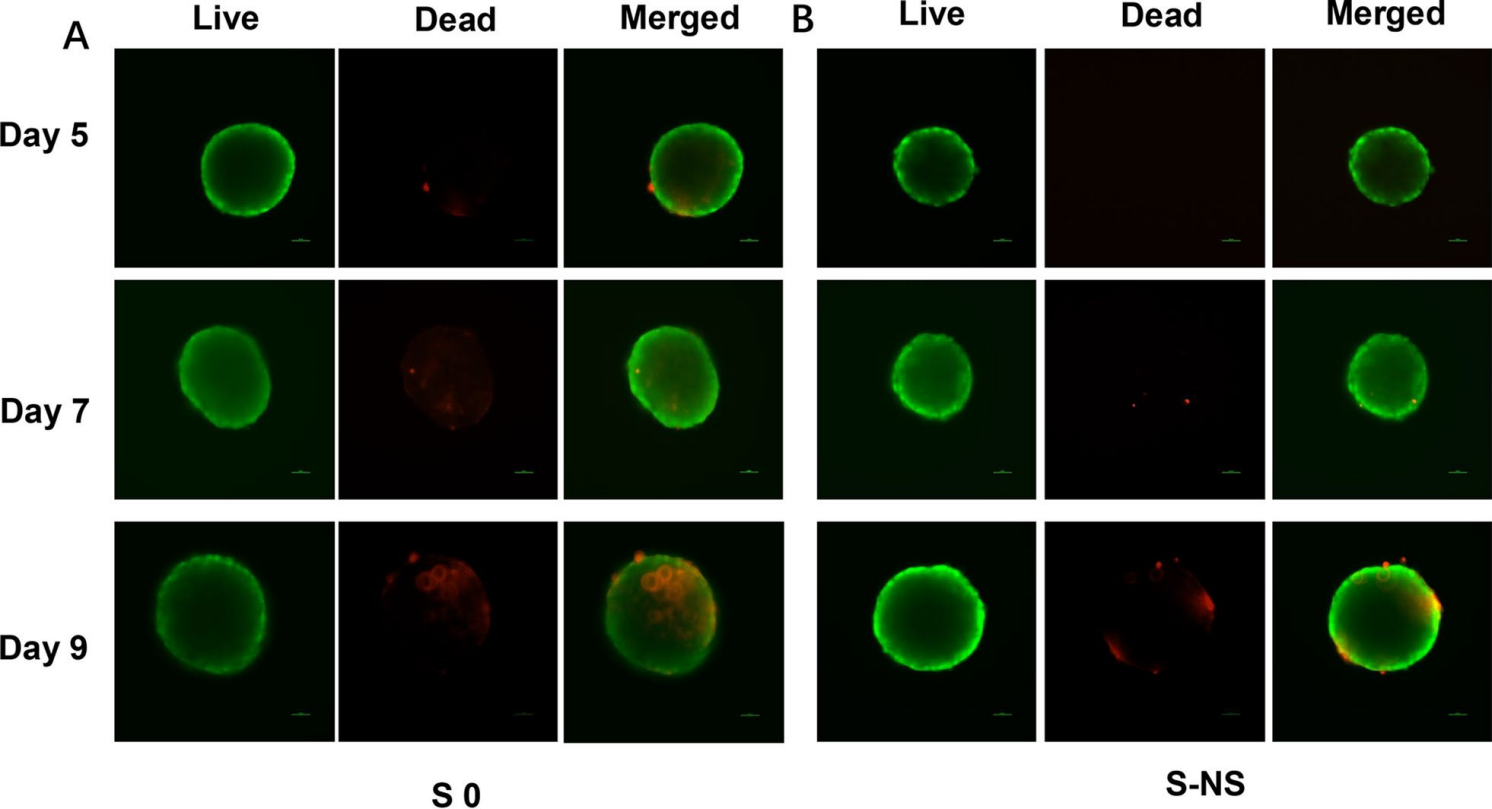
Live/dead staining of **A** S0 and **B** S NS-005 on days 5, 7, and 9. Objective: 20×. Scale bar: 50 μm. S0, spheroids without nanoscaffolds; S-NS, spheroids with nanoscaffolds

In our previous study, the cell viability during 11 days of culture was well assessed, and spheroids on day 5 were identified as optimal for drug-screening applications [24]. Therefore, this study focuses on evaluating the key time points relevant to the drug-screening process, i.e., days 5–9. Cell viability was evaluated by live/dead staining. As shown in Fig. 2, S0 displayed fluorescence signals associated with dead cells (red fluorescence) from day 5, and the intensity increased on the following days (day 7 and day 9). In contrast, for S-NS, only weak red fluorescence was observed from day 7, and there were fewer dead cells on day 9 compared with S 0, which indicated improved cell viability in scaffold-based spheroids. Notably, no evident necrosis was observed in S-NS groups within 9 days.

### The influence of nanoscaffolds on liver functionality

**Fig. 3:**
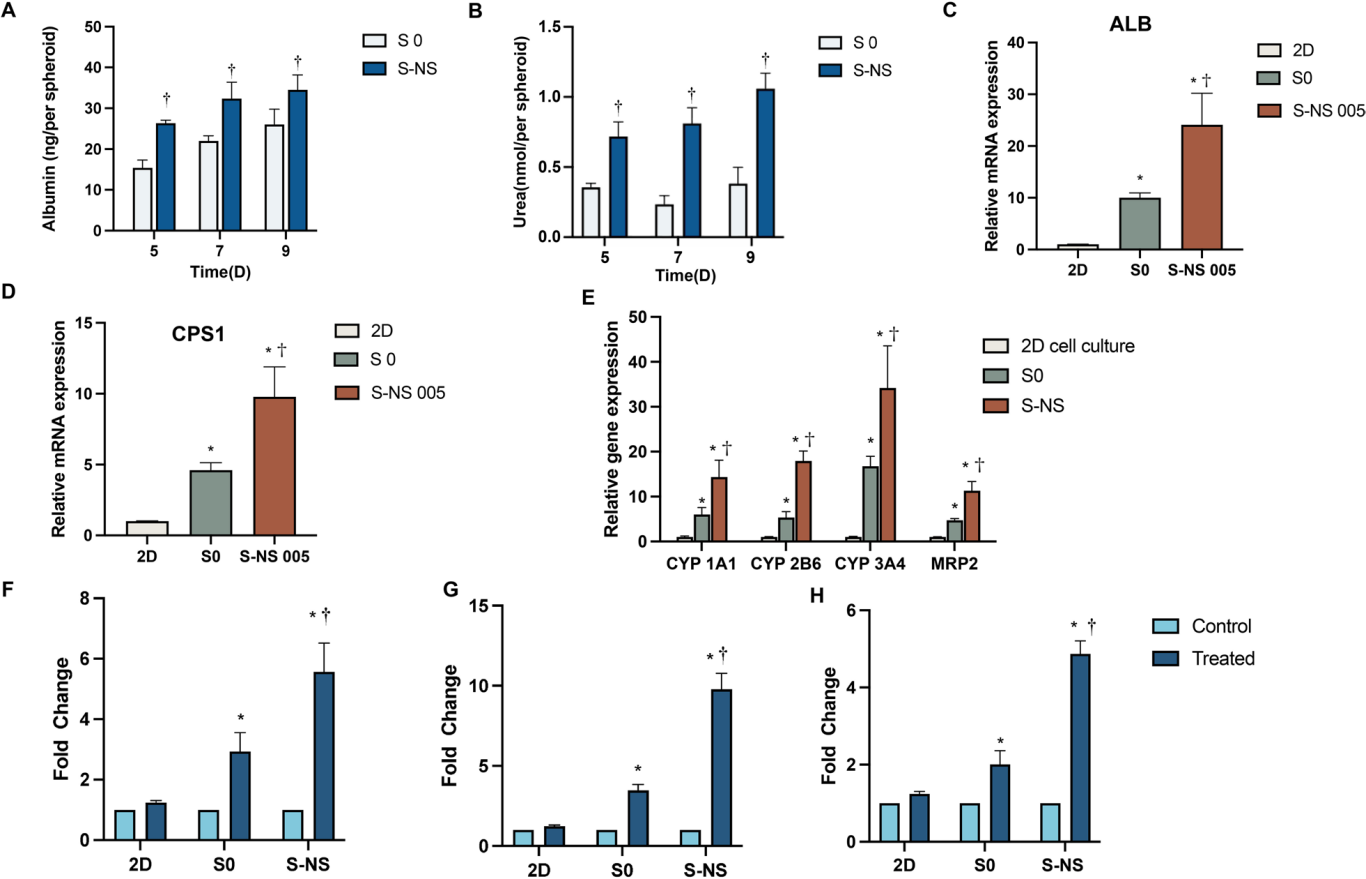
The phenotype of liver cells and drug-metabolism functions. **A** Albumin secretion (ELISA). **B** Urea secretion. qPCR analysis of the relative expression of **C** ALB and **D** CPS1. **E** qRT-PCR analysis of major drug metabolic gene expression, which was normalized to levels of *GAPDH*, and compared with cells cultured in the 2D condition. Enzyme activity of **F** CYP1A2, **G** CYP2B6, and **H** CYP3A4. *n* = 3, * *P* < 0.05 compared with 2D, ^†^*P* < 0.05 compared with S0. S0, spheroids without nanoscaffolds; S-NS, spheroids with nanoscaffolds; ALB, albumin; CPS1, carbamoyl phosphate synthetase 1

To establish a biologically relevant in vitro liver model, spheroids should be able to replicate the phenotypes and functionality observed in vivo. In this study, albumin and urea were used as biomarkers for liver cell phenotypes. As shown in Fig. 3A, B, the spheroids were able to maintain albumin and urea secretion during the 9-day culture period. The levels of albumin and urea were significantly higher in S-NS group than in the S0 group after 5 days of culture, which aligned with our previous results (*t*-test, albumin: day 5: *t*_(16)_ = 16.13, *P* < 0.0001; day 7: *t*_(16)_ = 7.374, *P* < 0.0001; and day 9: *t*_(16)_ = 4.881, *P* < 0.0001. Urea: day 5: *t*_(16)_ = 12.28, *P* < 0.0001; day 7: *t*_(16)_ = 23.95, *P* < 0.0001; and day 9: *t*_(16)_ = 22.87, *P* < 0.0001) [24]. In addition, we tested the expression level of relevant genes. Compared with 2D models, the expression of albumin (ALB) and urea cycle key enzyme CPS1 was significantly improved in S0 (tenfold and 4.6-fold, respectively) and S-NS (24.1-fold and 9.8-fold, respectively) (ANOVA: ALB: *F*_(2,15)_ = 64.39, *P* = 0.0117. CPS1: *F*_(2,15)_ = 74.88, *P* < 0.0001) (Fig. 3C, D).

Given that the liver plays a vital role in drug metabolism, it is essential to validate this functionality of the drug screening model. In this study, quantitative PCR (qPCR) was employed to evaluate the expression of major drug-metabolizing enzymes and transporters. Cytochrome P450 (CYP450) is the major enzyme family responsible for human drug metabolism, with CYP1A2, CYP2B6, and CYP3A4 playing key roles. As shown in Fig. 3E, after 5 days of culture, the mRNA expression of *CYP1A1*, *CYP2B6*, and *CYP3A4* in the S0 group were 6-fold, 5.3-fold, and 19.3-fold higher than in 2D groups, respectively. Furthermore, S-NS showed a further increase than S0, with a 17.7-fold, 17.9-fold, and 45.8-fold higher expression than in 2D groups, respectively. In addition, multidrug resistance-associated protein 2 (MRP2), which is a hepatic efflux transporter and also plays an important role in the formation of metabolites and drug detoxification, exhibited a higher expression level in S0 (4.7-fold) and S-NS (11.3-fold) groups compared with 2D cultures (ANOVA: CYP1A1: *F*_(2,15)_ = 76.69, *P* < 0.0001; CYP2B6: *F*_(2,15)_ = 214.1, *P* < 0.0001; CYP3A4: *F*_(2,15)_ = 53.11, *P* < 0.0001; and MRP2: *F*_(2,15)_ = 113.1, *P* < 0.0001).

To further evaluate the inducibility of drug-metabolism enzymes, we analyzed the enzyme activity of CYP1A2, CYP2B6, and CYP3A4 after treatment with their respective prototypical inducers (quinine, carbamazepine and rifampicin, respectively) in spheroids with and without nanoscaffolds. As shown in Fig. 3F–H, using the 2D cell culture model as a control, the activity of CYP1A2 in S0 and S-NS was induced by 2.9-fold and 5.6-fold, respectively (ANOVA: *F*_(2,6)_ = 33.29, *P* = 0.0006). For 2B6, the activity was increased by 3.5-fold and 9.8-fold, respectively (ANOVA: *F*_(2,6)_ = 154.3, *P* < 0.0001), while for CYP3A4, the activity was increased by 2-fold and 4.9-fold, respectively (ANOVA: *F*_(2,6)_ = 136.1, *P* < 0.0001). These results indicated that nanoscaffolds can sustain and enhance the specific functions of HepG2 spheroids.

### The influence of nanoscaffolds on cell–ECM interactions

**Fig. 4:**
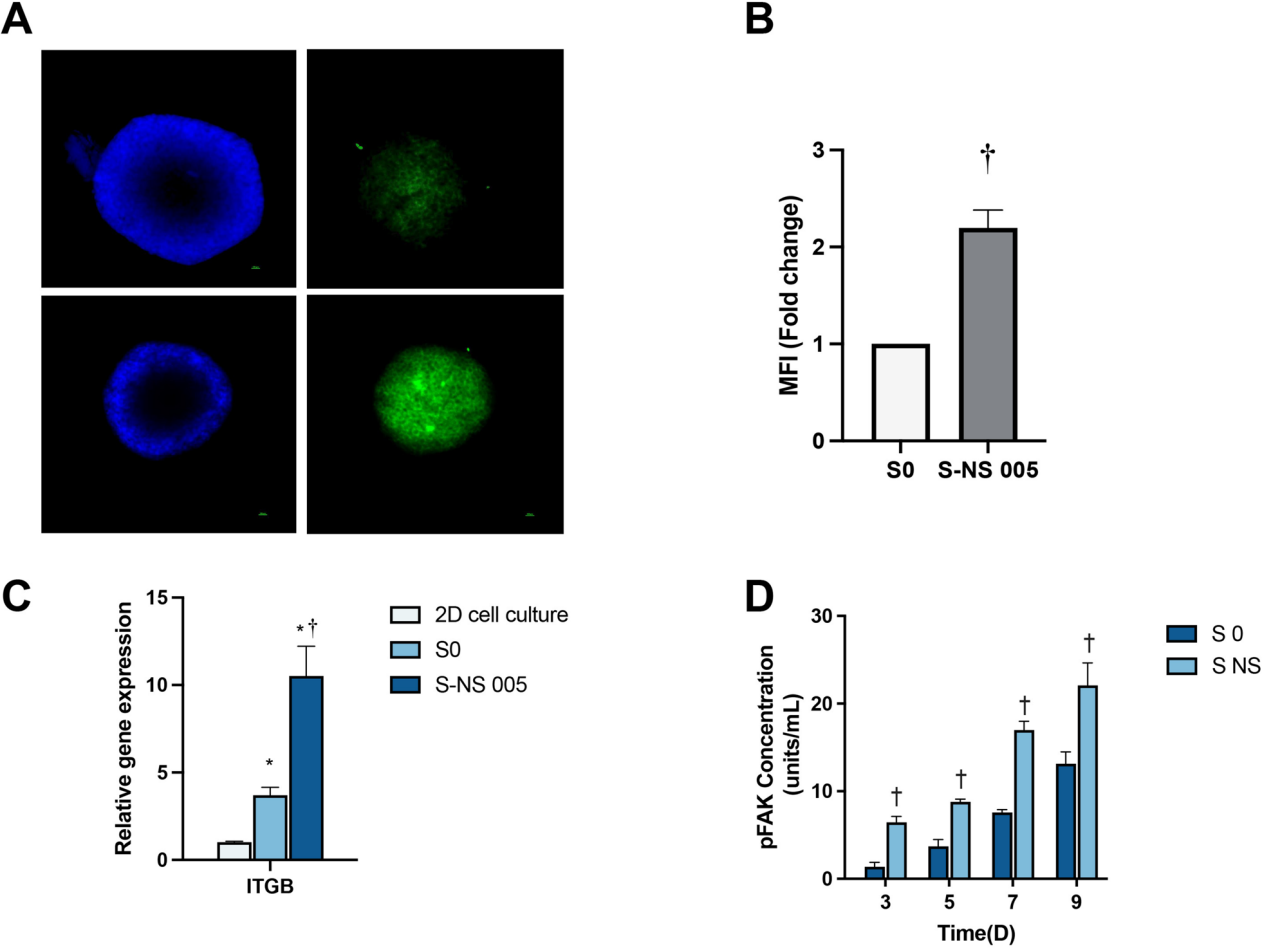
Analysis of cell–cell and cell–ECM junctions by: **A** immunofluorescence staining for integrin beta (ITGB), **B** mean fluorescence intensity (MFI) of ITGB fluorescence (images from three spheroids), **C** qPCR analysis of *ITGB* expression, and **D** pFAK level by ELISA. *n* = 3, * *P* < 0.05 compared with 2D, ^†^*P* < 0.05 compared with S0. S0, spheroids without nanoscaffolds; S-NS, spheroids with nanoscaffolds; FAK, focal adhesion kinase

The interaction between cells and the ECM plays a critical role in a plethora of cellular functions. ECM–integrin binding plays a key role in mediating these interactions. To evaluate the interaction of HepG2 spheroids with the nanoscaffolds as an artificial ECM, we employed immunostaining for integrin as a biomarker for cell–ECM interaction. Figure 4A, B, showed a notable stronger immunofluorescence staining signal of integrin beta (ITGB) in the S-NS group compared with the S0 group (*t*-test: *t*(16) = 21.91, *P* < 0.0001). qPCR results further confirmed these findings, as shown in Fig. 4C, with the expression levels of *ITGB* mRNA in S0 and S-NS being 3.7-fold and 10.5-fold higher than in 2D conditions, respectively (ANOVA: *F*_(2,15)_ = 140.7, *P* < 0.0001). We then evaluated the expression level of phosphorylated focal adhesion kinase (FAK) [pY397], which plays a pivotal role in transmitting signals triggered by integrin–ECM interactions into the cell, on days 5, 7, and 9 by ELISA. Figure 4D shows that FAK [pY397] level was significantly higher in S-NS compared with S0 over the 9-day measurement period (*t*-test, day 5: *t*_(10)_ = 15.10, *P* < 0.0001; day 7: *t*_(10)_ = 22.35, *P* < 0.0001;and day 9: *t*_(10)_ = 7.596, *P* < 0.0001).

### The effect of nanoscaffolds on drug penetration

**Fig. 5:**
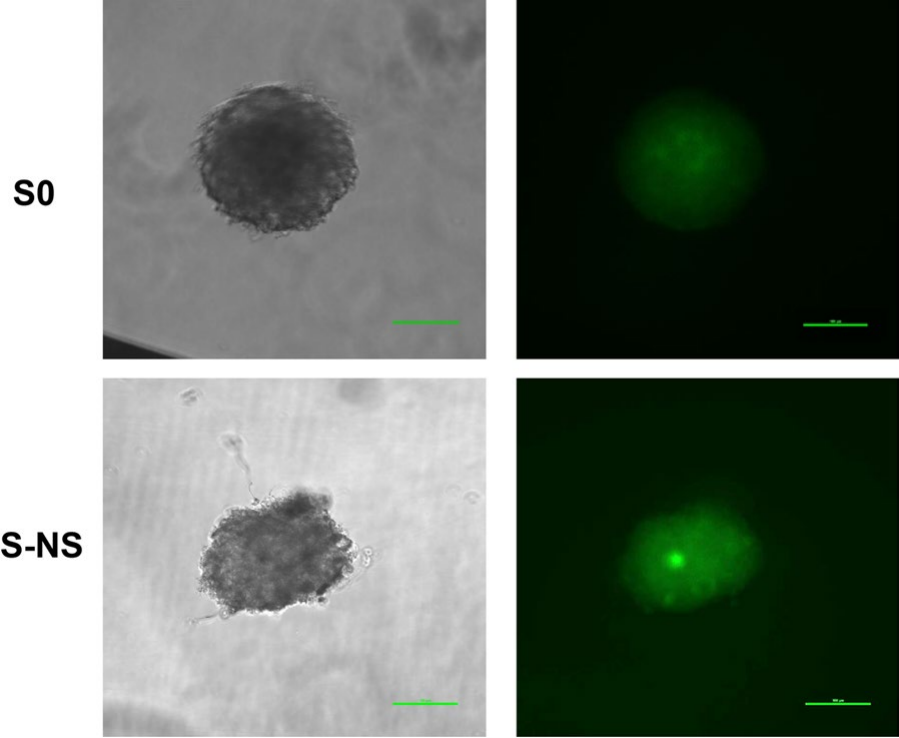
Drug penetration throughout S0 and S-NS. Scale bar = 100 μm. S0, spheroids without nanoscaffolds; S-NS, spheroids with nanoscaffolds

The uniform distribution of drugs in spheroids is important as hepatotoxicity models [31]. In order to confirm the effect of nanoscaffolds on drug penetration, doxorubicin was used here as a model drug. Doxorubicin is a lipophilic chemotherapeutic compound with intrinsic autofluorescence and is widely used for the study of drug penetration in spheroids [31–33]. We investigated the distribution of doxorubicin in S0 and S-NS. The penetration of doxorubicin in the spheroids was analyzed by fluorescence microscopy. As shown in Fig. 5, spheroids treated with 2 μM doxorubicin (green fluorescence) for 24 h exhibited homogeneous fluorescence signals distributed throughout both the peripheral and the central regions of the spheroids. Notably, comparable fluorescence distribution patterns were observed in both S0 and S-NS groups, confirming that the presence of the nanoscaffold did not hinder drug diffusion. On the basis of these findings, it was hypothesized that the majority of cells in the spheroids were exposed to comparable concentrations of compounds under the experimental conditions, making it suitable for drug toxicology studies.

### Hepatotoxicity evaluation of antimalarial drugs

**Fig. 6:**
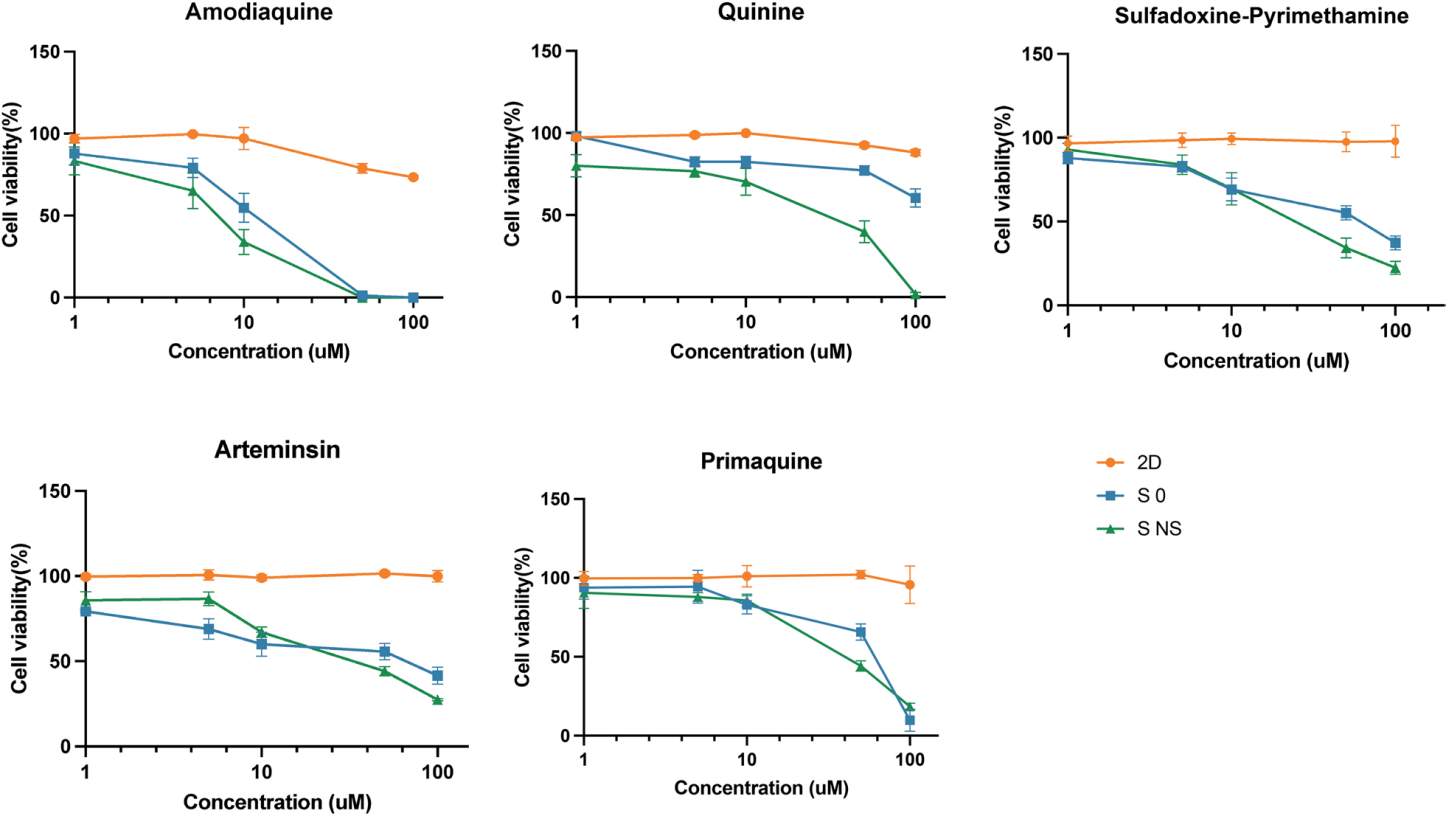
Cell viability analysis of spheroids with and without nanoscaffolds with the treatment of antimalarial drugs. 2D, two-dimensional models; S0, spheroids without nanoscaffolds; S-NS, spheroids with nanoscaffolds

To investigate the potential application of our scaffold-based spheroid system in assessing DILI, we evaluated the response of HepG2 cells to several known hepatotoxic antimalarial drugs (details in Table S2). Using 2D cell-culture models as controls, we found that HepG2 cells cultured in 3D spheroids exhibited significantly reduced viability upon treatment with amodiaquine (liver toxicity category A), quinine (category B), sulfadoxine/pyrimethamine (category C), artemisinin (category D), and primaquine (category E), where liver toxicity ranges from high (category A) to low (category E) (Fig. 6). Notably, the sensitivity of the S-NS group to drug-induced toxicity increased further compared with the S0 group, as indicated by lower IC_50_ values (Fig. 8A), suggesting that S-NS could better predict DILI. Moreover, the distribution of the IC_50_ values of the five drugs aligns with the known hepatotoxicity classifications in Livertox [34], indicating that our scaffold-based spheroid model system can sensitively identify the cytotoxicity of hepatotoxic agents for evaluating DILI.

### Hepatotoxicity evaluation of antimalarial candidate compounds

**Fig. 7:**
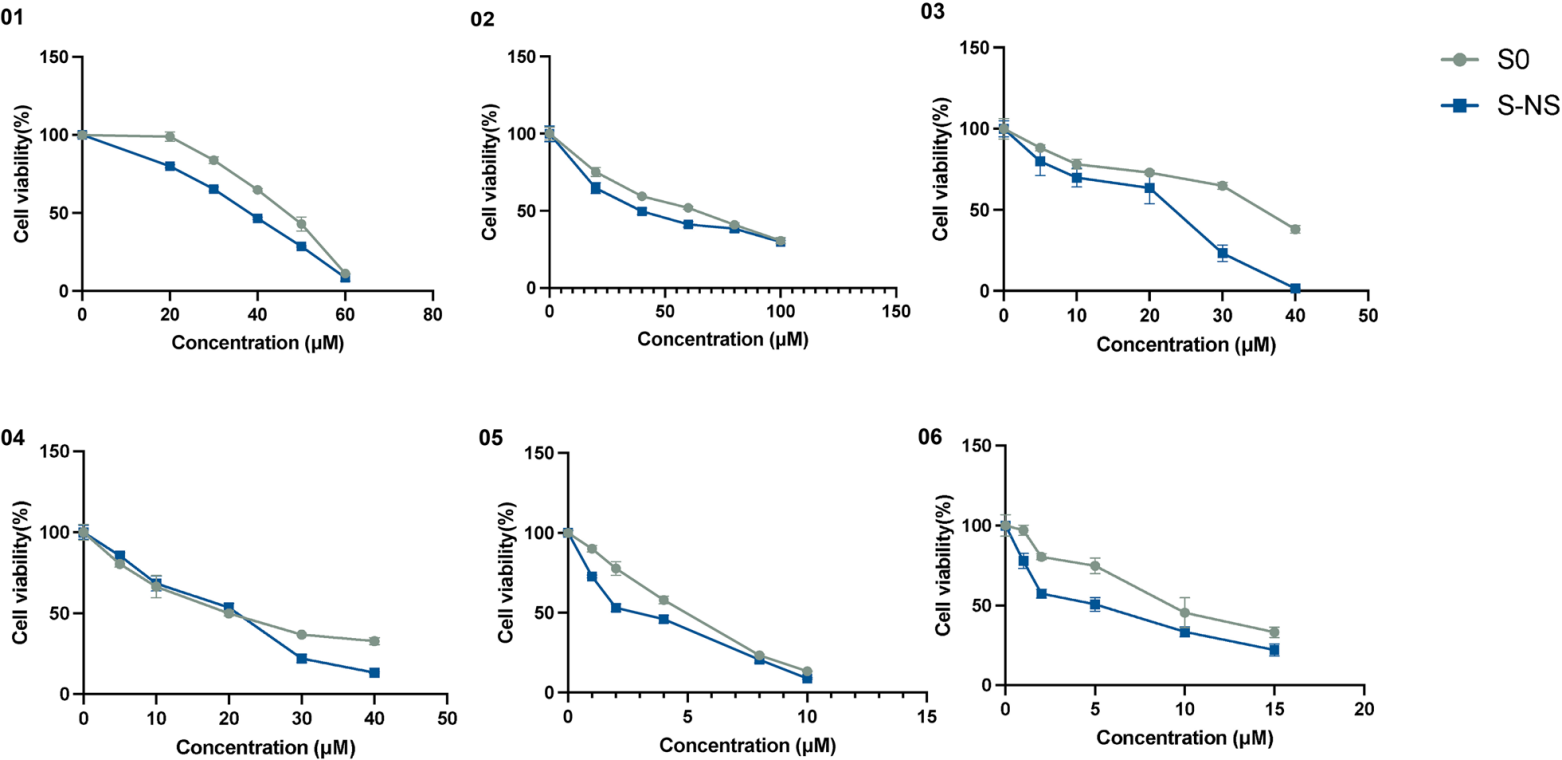
Cell viability analysis of spheroids with and without nanoscaffolds with the treatment of MMV compounds. The details of MMV compounds are listed in the Supporting Information. MMV, Medicines for Malaria Venture

**Fig. 8:**
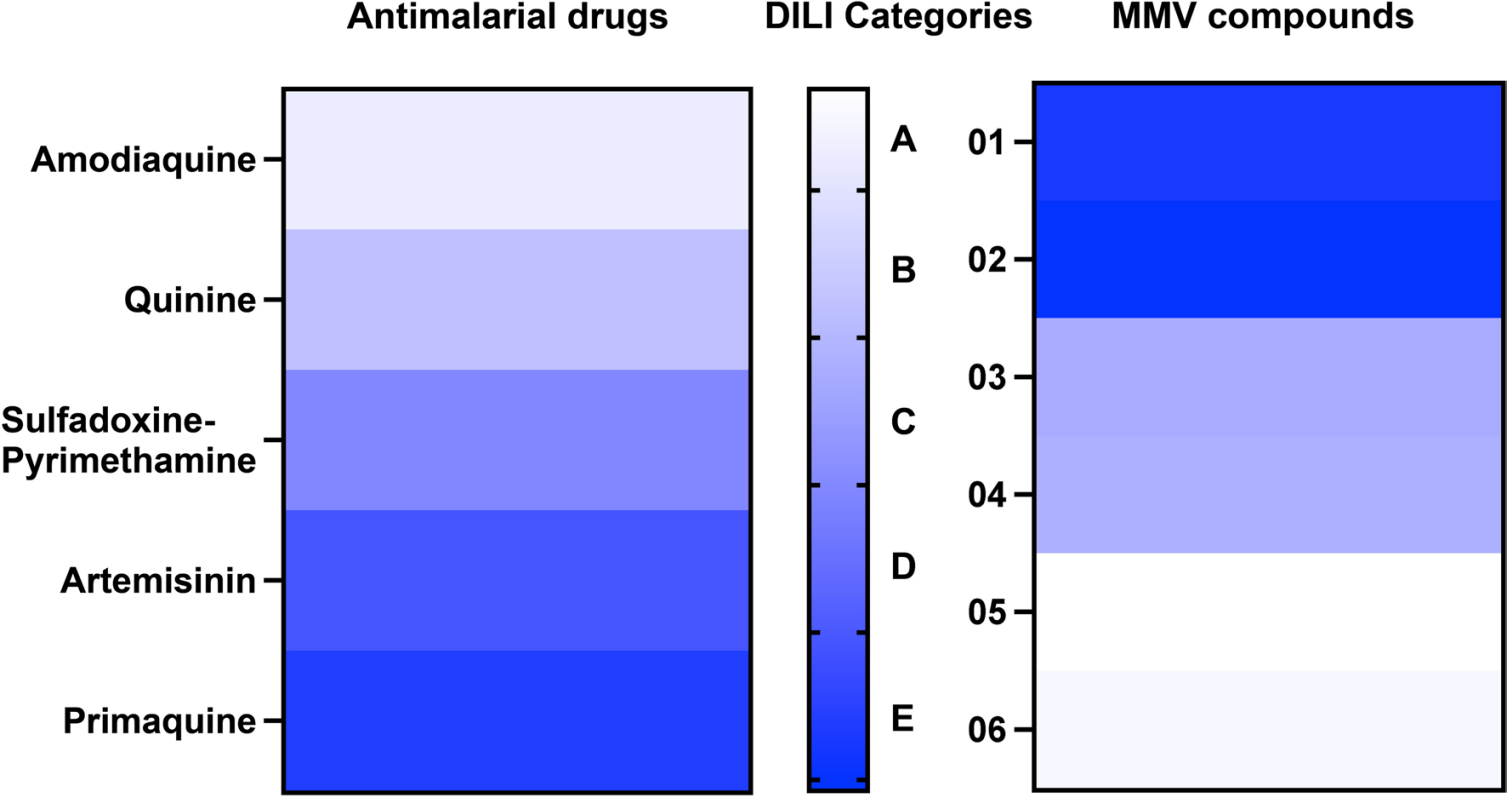
The DILI category map of antimalarial drugs and MMV compounds. DILI, drug-induced liver injury; MMV, Medicines for Malaria Venture

To further evaluate the applicability of our DILI model in preclinical drug candidates, the liver toxicity of six candidate compounds from MMV GHPB with antimalarial activity was evaluated. As shown in Fig. 7, both S0 and S-NS groups exhibited dose-dependent toxicity across all compounds. For the S-NS systems, sensitivity was notably higher, particularly with compounds 01, 02, 03, 05, and 06. Furthermore, the S-NS systems demonstrated lower IC_50_ values across all compounds compared with S0 (Table 2), indicating greater sensitivity to drug-induced hepatotoxicity. On the basis of the IC_50_ values of antimalarial drugs with established hepatotoxicity classifications, we generated a reference heatmap correlating IC_50_ values to hepatotoxicity levels using five distinct color codes (Fig. 8, left). Subsequently, the IC_50_ values of candidate drugs were input into this model, and their corresponding hepatotoxicity risk categories were assigned by matching their heatmap-derived color profiles to the reference scale. This comparative analysis enabled preliminary classification: compounds 01 and 02 were categorized as class E (lowest risk), compounds 03 and 04 as class B (moderate risk), and compounds 05 and 06 as class A (highest hepatotoxicity risk) (Fig. 8, right).

**Table 2:** IC*50* values for spheroids with and without nanoscaffolds

Drugs	IC_50_ (μM)		*P*-value
	S0	S-NS	
Antimalarial drugs			
Amodiaquine	14.73 ± 6.23	6.09 ± 0.58	0.0169^*^
Quinine	42.61 ± 1.01	15.34 ± 3.24	0.0024^*^
Sulfadoxine/pyrimethamine	52.89 ± 3.45	25.3 ± 1.33	0.0021^*^
Artemisinin	56.75 ± 2.75	32.4 ± 0.7	0.0027^*^
Primaquine	59.55 ± 3.39	37.16 ± 2.61	0.0011^*^
MMV compounds			
01	44.73 ± 0.87	39.58 ± 1.80	0.0113^*^
02	56.14 ± 3.14	29.54 ± 2.98	0.0039^*^
03	37.97 ± 1.07	17.40 ± 2.55	0.0018^*^
04	19.09 ± 1.77	9.73 ± 1.42	0.169
05	4.61 ± 0.54	3.44 ± 0.09	0.0205^*^
06	8.55 ± 0.52	4.29 ± 0.35	0.0006^*^

^*^ Statistically significant. Data are represented as mean ± SD, *n* = 3. S0, spheroids without nanoscaffolds; S-NS, spheroids with nanoscaffolds; MMV, Medicines for Malaria Venture

## Discussion

The liver is the most important organ for drug metabolism, with about 90% of drugs processed by cytochrome P450 enzymes in liver cells [35]. In addition, many drugs only exhibit therapeutic effects or toxicity after being metabolized by the liver [36]. Therefore, the liver is particularly sensitive to drug toxicity, and hepatotoxicity is a critical metric in toxicity testing in drug development [37]. A significant shortcoming of the use of traditional in vitro 2D cell culture models to predict in vivo response is that they fail to accurately replicate the complex physiology and functionality of the liver, leading to a lack of sensitivity to hepatotoxicity [18]. In vivo, cells within tissues and organs establish specific cell–ECM interactions, which are essential for their structural and biological behaviors. Furthermore, evidence showed that the cell–ECM interaction could also influence drug responses [38, 39].

Our previous research showed that electrospun PCL nanoscaffolds could significantly enhance the performance of HepG2 spheroids, with improved cell viability, albumin and urea secretion, cell–ECM interaction, and CYP450 activity. In addition, with acetaminophen as a model drug, we have demonstrated that our scaffold-based HepG2 spheroids system has the potential to be employed as a hepatotoxicity prediction tool [24]. In this study, we advanced the application of our scaffold-based liver spheroid system to evaluate the hepatotoxicity of antimalarial drugs, aiming to assess its broader potential in preclinical drug screening applications.

In this study, our results further validate the role of nanoscaffolds in improving the cell behavior in HepG2 spheroids. We found that compared with scaffold-free spheroids, the cell viability was improved in nanoscaffold-based spheroids. The spheroids with PCL nanoscaffolds could synthesize and secrete more albumin and urea than scaffold-free spheroids, and therefore could better represent the microenvironment of liver tissues. Furthermore, the expression of key genes related to albumin and urea secretion was significantly increased in scaffold-based spheroids, offering further evidence of improved liver phenotype.

Many drugs and compounds exhibit hepatotoxicity only after undergoing drug metabolism in the liver, with acetaminophen being a typical example [40]. Furthermore, studies have found that the antimalarial drug amodiaquine primarily metabolises into N-desethylamodiaquine (NADQ) through CYP1A1 and CYP3A4, which can induce apoptosis and lead to hepatotoxicity [41]. Among them, CYP3A4 is the most significant drug-metabolizing enzyme, metabolizing over 50% of clinically used drugs [42]. Research has shown that nearly half (49.1%) of the 3312 cases of DILI associated with CYP metabolism were attributed to CYP3A4/5. CYP1A1, CYP1A2, and CYP2D6 also play important roles in drug metabolism, particularly in the metabolism of antimalarial drugs [43, 44]. In addition, transporter families such as MRP2 (ABCC2) are also involved in phase III metabolism of antimalarial drugs including chloroquine [45] and amodiaquine [46]. The inhibition of these proteins will lead to an increased risk of DILI [25, 47] owing to the insufficient clearance of a drug or its metabolite, causing accumulation to cytotoxic levels that trigger DILI [25]. We demonstrated that HepG2 cells in nanoscaffold-based spheroids express key genes associated with drug-metabolizing enzymes and transport proteins, such as CYP1A2, CYP2B6, CYP3A4, and MRP2, with expression levels significantly higher than those in 2D cultures and scaffold-free spheroids, indicating that our spheroids possess drug metabolism capabilities and enable more accurate predictions of drug metabolism-related hepatotoxicity.

Certain drugs can influence the activities of specific CYP enzymes, with examples including quinine, an inducer of CYP1A2 [48], and amodiaquine, an inhibitor of CYP2D6 [49]. These could significantly impact the efficacy of drug–drug combinations. Therefore, it is crucial to understand the effects of antimalarial drugs on CYP enzyme activity since the treatments of malaria often involve the combination of multiple medications. When combination therapy is used, if antimalarial drugs induce the activity of specific CYP enzymes, it may accelerate the metabolism of other drugs, leading to reduced efficacy or potential toxic reactions [50]. Therefore, these interactions must be considered when developing treatment plans to optimize therapeutic outcomes and minimize side effects.

In addition, the expression of drug-metabolizing enzymes varies across different populations [51, 52]. For example, drug dosage guidelines are often based on data from white subjects, which may lead to hepatotoxicity in individuals from other ethnic groups. Unfortunately, only about 3% of clinical trial participants worldwide are of African genetic background [52]. This lack of representation results in limited clinical data on the safety and efficacy of drugs commonly used in African populations; additionally, studies have shown polymorphisms in the cytochrome CYP450 enzyme family, such as CYP2D6, can lead to variations in drug metabolism rates, which is a significant factor contributing to treatment failure in patients with malaria [53]. In addition, the expression of P450 enzymes in patients with malaria significantly differs from that observed in uninfected individuals. CYP2D6 and CYP2C19 metabolic phenotypes were inhibited in patients with *Plasmodium vivax* malaria [54]. Therefore, constructing personalized models that reflect different enzyme expression levels is crucial for achieving precision medicine. Overall, the inducibility of hepatic drug-metabolizing enzymes is critical when studying drug-induced liver toxicity. We found that after treatment with the relevant inducing agents, the activities of CYP1A2, CYP2B6, and CYP3A4 were significantly increased. The results demonstrated that our model has the potential for studying drug–drug interactions and developing personalized models with different enzyme activity levels.

Cell–ECM interaction is crucial for liver microstructure and liver function [55]. Due to weakened cell–ECM interactions, cells cultured in monolayers gradually lose their tissue-specific polarity [56]. Integrins, which are transmembrane cell adhesion receptors, have been shown to play an important role in mediating cell–ECM interactions, with integrin beta (ITGB) being one of the most important components [57]. When ITGB is inhibited, both cell–cell and cell–ECM junctions decrease, leading to reduced liver-specific gene expression and functional levels [58, 59]. Focal adhesion kinase (FAK) is a key component of the signal transduction pathways triggered by integrins. Upon integrin activation, FAK undergoes autophosphorylation at key tyrosine residues Y397 (pFAK) and mediates downstream signalling pathways of cell–ECM interaction [60]. In the present study, higher expressions of cell–ECM biomarkers (ITGB and pFAK) were observed in our scaffold-based spheroid models. These results strongly support that HepG2 cells in scaffold-based spheroids exhibit significantly enhanced cell–ECM connections, which may be a potential reason for the improvement in their liver-specific functions.

A notable limitation of 3D models compared with 2D systems is the poor drug penetration caused by enhanced cell–cell junctions, which often leads to heterogeneous drug distribution and reduced assay accuracy [61]. In our spheroid systems, both scaffold-free (S0) and scaffold-based (S-NS) spheroids showed distribution of the model drug in the central regions, which indicated that our 3D platform provides enhanced reliability for toxicity screening by ensuring consistent drug exposure across all cellular subpopulations within the spheroids.

In this study, we employed our nanoscaffold-based liver spheroid system to test the hepatotoxicity of five clinically approved antimalarial drugs (amodiaquine, quinine, primaquine, sulfadoxine/pyrimethamine, and artemisinin) and six compounds with antimalarial activity from the GHPB. The IC_50_ value, a widely recognized and reliable metric, was used as the main indicator for assessing drug-induced hepatotoxicity. We first assessed the IC_50_ values for each compound and found that our scaffold-based spheroids systems have lower IC_50_ values for all tested drugs than scaffold-free systems, indicating a higher sensitivity to liver toxicity. Therefore, they serve as effective models for drug toxicity screening. We then cross-referenced the IC_50_ values of the five clinically used antimalarial drugs, which have defined liver toxicity levels from the LiverTox classification system [34] as hepatotoxicity criteria, and made primary toxicity predictions for the candidate compounds. With this method, we can preliminarily associate the IC_50_ values of candidate drugs with their DILI levels, allowing us to predict the potential risk of hepatotoxicity during clinical use.

## Conclusions

This study demonstrates the translational application of our validated nanoscaffold-based 3D human liver spheroid model for in vitro hepatotoxicity assessment of antimalarial compounds at early-stage development and the post-marketing stage. Using clinically approved antimalarial drugs to establish a DILI reference framework, the model enabled the classification of candidate compounds from the GHPB according to their relative hepatotoxic risk. These findings highlight the value of this platform as a predictive, human-relevant, and high-throughput platform that can complement or replace animal testing in preclinical safety assessment, thereby supporting the development of safer and more effective antimalarial therapies.

## Supplementary Information

Additional file 1.

## Data Availability

The data supporting the main findings of this study are available within the manuscript and its Supplementary Information.

## Abbreviations

WHO: World Health Organization
DILI: Drug-induced liver injury
AQ: Amodiaquine
2D: Two-dimensional
3D: Three-dimensional
FDA: Food and Drug Administration
ECM: Extracellular matrix
PCL: Polycaprolactone
FBS: Fetal bovine serum
MEME: Minimum Essential Medium Eagle
NEAA: Nonessential amino acid solution
PBS: Phosphate-buffered saline
MMV: Medicines for Malaria Venture
GHPB: Global Health Priority Box
SEM: Scanning electron microscope
MRP2: Multidrug resistance-associated protein 2
ITGB: Integrin beta
FAK: Focal adhesion kinase
NADQ: N-desethylamodiaquine
CYP: Cytochrome P450
