# Supplementary material for: Nanoscaffold-based 3D human liver spheroids for predictive hepatotoxicity screening of antimalarial compounds from the global health priority box
Source: Parasit Vectors. 2026 Mar 23;19:194. doi: 10.1186/s13071-026-07324-1 (PMC13130545; doi:10.1186/s13071-026-07324-1)
Supplement: Supplementary file 1 — Additional file 1. [file 13071_2026_7324_MOESM1_ESM.docx]

**Additional File 1**

**Supporting Information**

**Nanoscaffold-Based 3D Human Liver Spheroids for Predictive Hepatoxicity Screening of Antimalarial Compounds from the Global Health Priority Box (GHPB)**

**Table S1** Primer sequences used

|  | **Forward (5’-3’)** | **Reverse (5’-3’)** |
| --- | --- | --- |
| GAPDH | GTCTCCTCTGACTTCAACAGCG | ACCACCCTGTTGCTGTAGCCAA |
| CYP1A2 | TCATCCTGGAGACCTTCCGACA | GCCACTGGTTTACGAAGACACAG |
| MRP2 | GCCAACTTGTGGCTGTGATAGG | ATCCAGACTGCTGTGGGACAT |
| CYP2B6 | TTAGGGAAGCGGATTTGTCTTG | GGAGGATGGTGGTGAAGAGAG |
| CYP3A4 | TGTAAAGAAACACAGATCCCCC | TCAGGCTCCACTTACGGTG |
| ALB | GATGAGATGCCTGCTGACTTGC | CACGACAGAGTAATCAGGATGCC |
| CPS1 | CTAGCCTGGATTACATGGTCACC | CCTCAAAGGTACGACCAATAGCC |
| ITGB | GGATTCTCCAGAAGGTGGTTTCG | TGCCACCAAGTTTCCCATCTCC |

**Table S2** Antimalarials used in this study (Adapted from LiverTox^1^)

| **Drugs:** | Liver toxicity category | Description |
| --- | --- | --- |
| Amodiaquine | A | The drug is well known, well described and well reported to cause either direct or idiosyncratic liver injury, and has a characteristic signature  More than 50 cases including case series have been described. |
| Quinine | B | The drug is reported and known or highly likely to cause idiosyncratic liver injury and has a characteristic signature  between 12 and 50 cases including small case series have been described. |
| Sulfadoxine-Pyrimethamine | C | The drug is probably linked to idiosyncratic liver injury, but has been reported uncommonly and no characteristic signature has been identified  the number of identified cases is less than 12 without significant case series. |
| Artemisinin | D | Single case reports have appeared implicating the drug, but fewer than 3 cases have been reported in the literature  No characteristic signature has been identified, and the case reports may not have been very convincing.  Drug can only be said to be a possible hepatotoxin and only a rare cause of liver injury. |
| Primaquine | E | Despite extensive use, no evidence that the drug has caused liver injury.  Single case reports may have been published, but they were largely unconvincing.  The agent is not believed or is unlikely to cause liver injury. |

Reference

1. *LiverTox: Clinical and Research Information on Drug-Induced Liver Injury*, Bethesda (MD): National Institute of Diabetes and Digestive and Kidney Diseases; 2012.
